# Control of Biogenic Amines in Food—Existing and Emerging Approaches

**DOI:** 10.1111/j.1750-3841.2010.01774.x

**Published:** 2010-09

**Authors:** Aishath Naila, Steve Flint, Graham Fletcher, Phil Bremer, Gerrit Meerdink

**Affiliations:** Authors Naila and Flint are with Inst. of Food Nutrition and Human Health, Massey Univ.Private Bag 11-222 Palmerston North, NZ; Author Fletcher is with Food Safety & Preservation, New Zealand Inst. for Plant & Food Research LimitedPrivate Bag 92169, Auckland, NZ; Author Bremer is with Dept. of Food Science, Univ. of OtagoPO Box 56, Dunedin, NZ; Author Meerdink is with Dept. of Food Manufacture and Process Technology, Univ. of Lincoln—Holbeach CampusPark Rd., Holbeach PE12 7PT, U.K.

**Keywords:** biogenic amines, food additives, high hydrostatic pressure (HHP), irradiation, packaging, scombroid poisoning, starter cultures, temperature

## Abstract

Biogenic amines have been reported in a variety of foods, such as fish, meat, cheese, vegetables, and wines. They are described as low molecular weight organic bases with aliphatic, aromatic, and heterocyclic structures. The most common biogenic amines found in foods are histamine, tyramine, cadaverine, 2-phenylethylamine, spermine, spermidine, putrescine, tryptamine, and agmatine. In addition octopamine and dopamine have been found in meat and meat products and fish. The formation of biogenic amines in food by the microbial decarboxylation of amino acids can result in consumers suffering allergic reactions, characterized by difficulty in breathing, itching, rash, vomiting, fever, and hypertension. Traditionally, biogenic amine formation in food has been prevented, primarily by limiting microbial growth through chilling and freezing. However, for many fishing based subsistence populations, such measures are not practical. Therefore, secondary control measures to prevent biogenic amine formation in foods or to reduce their levels once formed need to be considered as alternatives. Such approaches to limit microbial growth may include hydrostatic pressures, irradiation, controlled atmosphere packaging, or the use of food additives. Histamine may potentially be degraded by the use of bacterial amine oxidase or amine-negative bacteria. Only some will be cost-effective and practical for use in subsistence populations.

## Introduction

Biogenic amines and polyamines have been reported in variety of foods, such as fish, meat, cheese, vegetables, and wines, and are described as organic bases with aliphatic, aromatic, and heterocyclic structures (Lorenzo and others 2007). Biogenic amine formation through the microbial decarboxylation of amino acids is dependent on the specific bacterial strain(s) present, the level of decarboxylase activity, and the availability of the amino acid substrate (Suzzi and Gardini 2003; Rivas and others 2008). Histaminolytic (histamine oxidizing) bacteria may allow an equilibrium to develop between histamine production and destruction in foods containing high amounts of histamine (Lenistea 1971). The most common biogenic amines found in foods are histamine, tyramine, cadaverine, 2-phenylethylamine, spermine, spermidine, putrescine, tryptamine, and agmatine. In addition octopamine and dopamine have been found in meat and meat products and fish (Hernandez-Jover and others 1996). Polyamines, such as putrescine, cadaverine, agmatine, spermine, and spermidine, are naturally present in food and are involved in growth and cell proliferation (Hernandez-Jover and others 1997; Kalac 2009; Kim and others 2009). These amines in the presence of nitrites can be potential carcinogens when converted to nitrosamines (Kim and others 2009). Nitrosamines from polyamines may not necessarily pose a health risk as toxicity is reached only after consumption of large amounts, more than expected in a daily meal (Kalac 2009). The aromatic biogenic amines, tyramine, and 2-phenylethylamine have been reported to be initiators of dietary-induced migraine and hypertensive crisis (Stratton and others 1991). Tyramine, 2- phenylethylamine, and putrescine are versoactive amines and increase blood pressure that can lead to heart failure or brain hemorrhage (Til and others 1997; Kalac 2009; Mohan and others 2009).

Histamine poisoning (scombroid poisoning) is a worldwide problem (Russell and Maretic 1986) that occurs after the consumption of food containing biogenic amines, particularly histamine at concentrations higher than 500 ppm (Gonzaga and others 2009). Histamine poisoning manifests itself as an allergen-type reaction characterized by difficulty in breathing, itching, rash, vomiting, fever, and hypertension. People having deficient natural mechanisms for detoxifying biogenic amines through genetic reasons or through inhibition due to the intake of antidepression medicines, such as monoamine oxidase inhibitors (MAOIs) are more susceptible to histamine poisoning (Hernandez-Jover and others 1997; Yongmei and others 2009). Histamine alone may not cause toxicity at a low level, but the presence of other biogenic amines such as putrescine and cadaverine, at concentrations 5 times higher than histamine, enhance the toxicity of histamine (Stratton and others 1991; Hernandez-Jover and others 1997; Emborg and Dalgaard 2006) through the inhibition of histamine oxidizing enzymes. Oral toxicity levels for putrescine, spermine, and spermidine are 2000, 600, and 600 ppm, respectively. The acute toxicity level for tyramine and cadaverine is greater than 2000 ppm. The no observed adverse effect level (NOAEL) is 2000 ppm for tyramine, putrescine, and cadaverine; 1000 ppm for spermidine; and 200 ppm for spermine (Til and others 1997). Tyramine alone at high levels can cause an intoxication known as the cheese reaction, which has similar symptoms to histamine poisoning.

When legumes were boiled, the biogenic amines transferred completely into the boiling water so by this means any hazard could be eliminated. However, sprouted legumes behaved differently when boiled, with the biogenic amine concentration being only reduced slightly (Shalaby 2000). This indicates that although biogenic amines in some type of legumes can be eliminated through boiling; boiling is not effective in eliminating biogenic amines in sprouted legumes. The effectiveness of biogenic amines elimination via boiling on other food products, each type requires experimentation. However, biogenic amines are reported as heat stable compounds (Tapingkae and others 2010) and cooking or prolonged exposure to heat will not eliminate the toxin (Shalaby 1996; Duflos 2009; Gonzaga and others 2009).

Factors influencing biogenic amine production are storage conditions (Komprda and others 2001), manufacturing processes (Rivas and others 2008), manufacturing practices (Komprda and others 2001) the proportion of the microbial population with decarboxylase activity (Santos 1996), raw material quality (Maijala and others 1995b), and the availability of free amino acids (Maijala and others 1995a).

Biogenic amines can be controlled with the use of existing methods and emerging methods. The existing method in this paper refers to temperature, which has been well established in control of biogenic amines. The emerging method in the paper refers to other methods to date used for controlling or eliminating biogenic amines in food than temperature or with the combination of temperature. The emerging methods include modified atmosphere packaging (MAP), irradiation, high hydrostatic pressure (HHP), and microbial modeling and addition of preservatives.

Biogenic amine formation can be controlled through inhibiting microbial growth or inhibiting the decarboxylase activity of microbes (Wendakoon and Sakaguchi 1995). The prevention of biogenic amine formation in food has, therefore, been achieved using temperature control, using high-quality raw material, good manufacturing practice, the use of nonamine forming (amine-negative) or amine oxidizing starter cultures for fermentation (Dapkevicius and others 2000; Nieto-Arribas and others 2009), the use of enzymes to oxidize amines (Dapkevicius and others 2000), the use of microbial modeling to assess favorable conditions to delay biogenic amine formation (Neumeyer and others 1997; Emborg and Dalgaard 2008a, 2008b), packaging techniques (Mohan and others 2009), HHP (Bolton and others 2009), irradiation (Kim and others 2003), and food additives (Mah and Hwang 2009a). Emerging approaches to control histamine production involve the combined effect of an existing method, such as the combination of HHP and amine-negative starters (Latorre-Moratalla and others 2007). However, optimization of such an approach is required.

This review will identify and discuss techniques that can be used to limit amine formation or enhance their degradation.

## Existing Methods for Biogenic Amine Control in Food

Biogenic amine formation is temperature dependent (Shalaby 1996), and it is decreased at low temperatures (Duflos 2009; Prester and others 2009) through inhibition of microbial growth and the reduction of enzyme activity (Arnold and others 1980; Chander and others 1989; Bremer and others 1998; Du and others 2002; Mah and Hwang 2009b). Biogenic amine formation in food can, therefore, be controlled by strict adherence to the cold chain (Bremer and others 2003; Bover-Cid and others 2006; Dalgaard and others 2006). Biogenic amine forming bacteria such as *Morganella morganii* and *Proteus vulgaris* in skipjack tuna (*Katsuwonus pelamis*) were inhibited through chilling (Arnold and others 1980; Ruiz-Capillas and Jiménez-Colmenero 2004). Maintaining the cold chain in foods that already contain high levels of biogenic amines will generally stabilize the levels of biogenic amines, although in some cases there may be a slight increase over time (Gonzaga and others 2009; Chen and others 2010). For example, yellowfin tuna stored at 0 °C and 22 °C up to 9 d, showed an increase in histamine of 15 ppm at 0 °C and 4500 ppm at 22 °C (Du and others 2002). Freezing is more effective than cooling in preventing biogenic amine production (Arnold and Brown 1978).

High-temperature treatments can also be used to extend the shelf life of food. A thermal regime designed to kill the bacterial species responsible for histamine formation and can prevent the subsequent formation of histamine. For a *Hafnia alvei* strain implicated in histamine production in hot smoked Kahawai, at temperatures between 54 and 58 °C, the D-values (the time required to kill 90% of the contaminating bacteria) ranged from 51 to 20 s (Bremer and others 1998). For *M. morgani* in hot smoked Kahawai at temperatures between 58 and 62 °C, the D-values were between 15 and 1.5 s (Osborne and Bremer 2000). Although heating can destroy the histamine-producing bacteria in food, if recontamination and temperature abuse occurs after thermal processing, histamine formation may still occur in the thermally processed product.

However, as discussed above histamine is heat stable so applying heat after histamine has formed in the product will not ensure its safety. For example, fish paste (Rihaakuru, Maldives local dish) is made through prolonged cooking (maximum 100 °C), which eliminates all the potential bacteria responsible for histamine formation. However, Rihaakuru often contains high levels of histamine (>1000 ppm) (authors unpublished data) as the histamine is believed to be formed in fish well before the cooking step and heat does not destroy histamine.

## Emerging Methods for Biogenic Amine Control

It is not always possible to control biogenic amine production through temperature alone, since some bacteria produce biogenic amines at temperatures below 5 °C (Emborg and others 2005; Emborg and Dalgaard 2006). In addition, in some societies, refrigeration is not readily available. In such circumstances, emerging methods of control need to be considered however, little work has been done on these.

Emerging methods as control measures include the addition of starter cultures that degrade histamine, the application of hydrostatic pressures, irradiation, packaging, using food additives and preservatives, and altering conditions based on microbial modeling of histamine producing bacteria. The majority of these methods are not new in terms of food preservation but are not commonly used in controlling biogenic amines. The use of enzymes, such as diamine oxidase (DAO) that degrade biogenic amines, and the use of bacteria that posses this enzyme, are the only potential tools to degrade already formed biogenic amines and are not currently recognized preservation methods.

The formation of biogenic amines is associated with food spoilage, suggests poor hygienic practices, and may therefore indicate other food safety issues. Any attempts to control biogenic amines must take into account the factors leading to the formation of the biogenic amine and ensure other food safety issues are not being overlooked. Products where a secondary control approach is justified are those that are microbiologically stable. An example is the fish paste product from the Maldives, Rihaakuru, with a maximum water activity of 0.8. Temperature abused fish, which has been rejected from fish factories, is used as the raw ingredient for Rihaakuru—a product made through prolonged cooking, that once produced, is stable at ambient temperature (25 to 30 °C) for over a year. Although Rihaakuru has nutritional benefits, rich in protein and omega 3 sources, a health concern is potential for scombroid poisoning due to high biogenic amines contents. Maintaining the cold chain is not a practical solution due to the cost of refrigeration being out of reach for the artisan fishers. One option to ensure the safety of products such as Rihaakuru is to destroy the biogenic amines in the product, but this has not been investigated. Most approaches to control histamine in a food such as Rihaakuru focus on delaying biogenic amine formation (Joosten and Nunez 1996; Fletcher and others 1999; Emborg and Dalgaard 2008a). Methods to destroy biogenic amines, particularly histamine, have not been seriously considered, as the sensory quality of foods with high biogenic amines is often unacceptable and biogenic amines are actually used as a freshness indicator in many foods (Pons-Sanchez-Cascado and others 2006). However, with Rihaakuru, the final product is microbiologically stable and biogenic amines formed in the fish before processing do not appear to be associated with sensory defects in the final product. The concern with histamine in Rihaakuru is food safety and prevention through normal handling through refrigeration of raw fish is impractical. This review also examines options for to the destruction of histamine in microbiologically stable foods such as Rihaakuru.

## Methods for Delaying Biogenic Amines Accumulation

### Application of food additives and preservatives

Additives and preservatives can reduce the formation of biogenic amines (Table 1) in products such as mackerel by inhibiting bacterial growth and amine formation (Kang and Park 1984). Sodium sorbate may limit the formation of biogenic amines and sodium hexametaphosphate at 2% has been shown to delay histamine production (Kang and Park 1984; Shalaby and Rahman 1995; Shalaby 1996). Citric acid, succinic acid, D-sorbitol, and malic acid inhibited decarboxylase activity and the resulting histamine formation in mackerel stored for 10 d at 25 °C (Shalaby 1996). Citric acid use (1%) during pickled cabbage fermentation produced a slight decrease in biogenic amines at a salt level of 6, 8, or 10% (Yuecel and Ueren 2008).

**Table 1:** Biogenic amines reduction through food preservatives.

**Food type**	**Additives applied**	**Storage condition**	**Storage time**	**Reduction in the formation of biogenic amines**	**Reference**
Meat	GDL; 0%, 0.5%, 1.0%	20 to 22 °C	7 d	Histamine (dropped from 126 to 7 ppm) and putrescine (dropped from 236 to 147 ppm)	(Maijala and others 1993)
Indian mackerel (whole)	10% (weight of fish) Curcumine (turmeric), capsaicin (red pepper), piperine (black pepper)	5 °C	8 d	All spices reduced biogenic amines (histamine (dropped from >200 to 13ppm), cadaverine (approximately dropped from 200 to 100 ppm) putrescine (approximately dropped from 100 to 25 ppm), and tyramine (approximately from 200 to <100 ppm)	(Shakila and others 1995)
Slightly fermented sausages	Sugar (glucose, lactose) between 4000 and 20000 ppm	4 °C and 19 °C	20 d	Cadaverine	(Bover-Cid and others 2001a)
				Tyramine	
Fermented sausage (Sucuk)	Potassium pyrophosphate (2500 ppm), di-potassium hydrogen phosphate (2500 ppm), ascorbic acid (500 ppm), alpha-tocopherol (200 ppm), potassium sorbate (200 ppm)	Temperature: 20 °C, 30 °C, and 40 °C % relative humidity (RH): 50, 65, and 80	60 d	Histamine (dropped from 242 to 35 ppm at 80% RH and at 30 °C), putrescine (dropped from 378 to 12 ppm at 65% RH and at 40 °C), tryptamine (dropped from 60 to 14 ppm at 50% RH and at 20 °C)	(Bozkurt and Erkmen 2004)
Myeolchi-Jeot (fermented anchovies)	5% garlic extract (dissolved in ethanol)	25 °C	10 wk	Histamine and tyramine reduced by 20.8% and 31.2%, respectively. Overall amines reduced by 8.7% compared with the control	(Mah and others 2009)
Myeolchi-Jeot (fermented anchovies)	5% glycine (weight basis), NaCl (20%)	25 °C	10 wk	Biogenic amines (putrescine, cadaverine, histamine, tyramine, spermidine) reduced between 63% and 73% compared with the control	(Mah and Hwang 2009)

Potassium sorbate has also been found to extend the shelf life of seafood (Shalini and others 2001). Sausage containing potassium sorbate, and ascorbic acid showed a significant reduction in biogenic amine accumulation (Bozkurt and Erkmen 2004). Sodium nitrites (45 to 195 ppm) in sausage decreased biogenic amine production, (Kurt and Zorba 2009). This confirms the findings of Bozkurt and Erkmen (2004) that sodium nitrite and sodium nitrate inhibit biogenic amine production. The addition of 0 to 1% glucono-delta-lactone (GDL) into meat decreased histamine and putrescine production through a pH drop in meat (Maijala and others 1993). The addition of sugar may also slightly reduce biogenic amine formation (Bover-Cid and others 2001a). When glycine was applied to Myeolchi-jeot, (a salted and fermented anchovy product) the overall production of biogenic amines was reduced by 63 to 73%. The authors concluded that glycine inhibits the amine forming activity of microorganisms. Biogenic amines in other fermented fish products may be reduced using glycine as a food additive (Mah and Hwang 2009a).

Naturally occurring specific inhibitory substances in spices and additives have also been shown to inhibit biogenic amine formation (Komprda and others 2004). Such substances include curcumin (turmeric), capsaicin (red pepper), and piperine (black pepper) (Wendakoon and Sakaguchi 1992; Shakila and others 1995). The disadvantage of these substances is the considerable loss in efficacy that occurs during cooking (Suresh and others 2007). Among these substances, capsaicin was found more heat stable than curcumin and piperine (Srinivasan and others 1992). However, capsaicin is a pungent component and excites primary sensory nurons (Someya and others 2003). The most active component of turmeric is curcumin, an analog of 6-gingerol. The turmeric yellow color appearance is due to curcuminoids. Curcumin has been used as a food additive, spice, and as a medicinal herb (Bhutani and others 2009). Curcumin levels of 8 g/d may be tolerable with approximate consumption being 0.1 g/d. It is a potent antioxidant 10 times more powerful than vitamin E (Shishodia and others 2005).

Components of spices, such as thymol may inhibit biogenic amine formation (Singh and others 1999). Thymol is a phenolic monoterpene, naturally found in essential oils, that has antioxidant and antimicrobial properties. It is a major component of thyme and oregano (Lee and others 2008). However, thymol, having unpleasant pungent flavor, may not be accepted by consumers as an ingredient for food formulation (Lee and others 2008).

Ginger, garlic, green onion, red pepper, clove, and cinnamon have been shown to delay biogenic amine production in Myeolchi-jeot (Mah and others 2009). The addition of 5% garlic during Myeolchi-jeot ripening reduced the biogenic amine level by 8.7% (Mah and others 2009). Garlic is one of the most popular herbs in the world used as a flavoring agent in food. Allicin is the most active ingredient in garlic, formed from allin by enzyme allinase when the garlic clove is crushed (Batra and Rajeev 2007). Ginger, lowers blood pressure, may cure hypertension and palpitations (Ghayur and others 2005), and it possess antibacterial and antifungal activity (Chrubasik and others 2005). The 6-gingerol, pungent constituent of ginger (Young and others 2005), is known to enhance gastrointestinal transport (Batra and Rajeev 2007). The 6-gingerol also been shown to have some inhibitory effect on biogenic amine formation (Singh and others 1999).

The effect of spices has been measured on specific bacteria that produce biogenic amines. Ethanol extracts of allspice, sage, cloves, cinnamon, and nutmeg were found to delay biogenic amine formation by *Enterobacter aerogenes*. The inhibitory effect was improved with the addition of sodium chloride (NaCl). Cinnamic aldehyde, a component of cinnamon, and eugenol, a compound of cloves were found to be the most effective inhibitors of biogenic amine formation by specific bacteria, *E. aerogenes* (Wendakoon and Sakaguchi 1995). Histamine formation by *M. morganii* was delayed in the presence of 0.5% potassium sorbate (Shalaby 1996) and by the essential oil of lemongrass (Sangcharoen and others 2009). Histamine formation in *Klebsiella pneumonia* was delayed by sorbate at 0.5% (Shalaby 1996). *Bacillus licheniformis,* an isolate from Myeolchi-jeot, is a strong biogenic amine former. Glycine (10%) was shown to reduce the histamine, cadaverine, and putrescine of *B. licheniformis* by 93, 78, and 32%, respectively, and reduce tyramine and spermidine production by 100% (Mah and Hwang 2009a).

Although studies have shown the inhibitory effects of food additives and preservatives on biogenic amine accumulation, few authors have highlighted their potential negative effects. For example, the presence of preservatives has been reported to increase biogenic amine formation during sausage production (Komprda and others 2004). Recently, it was found that curcumin inhibits DAO (Bhutani and others 2009), which may inhibit biogenic amine reduction. When sodium sorbate and sodium hexametaphosphate were applied to sardines, a putrefactive odor was observed within 2 d at chill storage (Kang and Park 1984). Other disadvantages of preservatives use are a lack of available knowledge on their effectiveness against biogenic amines in foods and the lack of consumer acceptance (Bjornsdottir 2009).

In summary, food additives and preservatives that work well in food require further investigation into the effectiveness in delaying biogenic amine production. Food additives that have shown a positive effect on delaying biogenic amine formation need to be tested in variety of food systems.

### High hydrostatic pressure

HHP is a nonthermal preservation method that damages cell membranes of microorganisms resulting in inactivation or sublethal injury (Rivas and others 2008). Through inactivation of microorganisms, HHP extends shelf life while retaining the original flavor and characteristics of food (Patterson 2005). HHP-treated foods are commercially available in the United States (for example, guacamole, oysters), Japan (for example, fruit jam), and Spain (for example, cooked and vacuum packed ham) (Patterson 2005). HHP has been applied to many other foods including cheese (Novella-Rodriguez and others 2002), sausage (Latorre-Moratalla and others 2007; Ruiz-Capillas and others 2007), fish (Bolton and others 2009), and sauerkraut (Peñas and others 2010).

When HHP is applied to raw material or the end products of fermentation, a reduction in the number of bacteria may inhibit biogenic amine formation (Table 2). For example, when HHP (200 MPa) was applied to meat batter raw material for sausage fermentation, it inhibited the growth of *Enterobacteria* and simultaneously delayed the accumulation of putrescine and cadaverine (Latorre-Moratalla and others 2007). Inhibition of biogenic amine formation depends on the level of pressure applied. For instance, during cheese ripening, a low-pressure treatment of 50 MPa for 72 h increased biogenic amine content, while a high-pressure treatment of 400 MPa for 5 min plus 50 MPa for 72 h showed a slight decrease (Novella-Rodriguez and others 2002).

**Table 2:** Biogenic amines reduction through high hydrostatic pressure.

**Food type**	**HHP applied**	**Storage condition**	**Storage time**	**Reduction in the formation of biogenic amines**	**Reference**
Goat cheese ripening	400 MPa for 5 min and 50 MPa for 72 h at 14 °C	Ripened at 14 °C and 86% RH	28 d	Tyramine dropped from 10.3 to 1.6 ppm	(Novella-Rodriguez and others 2002)
Meat batter, raw material for sausage fermentation	200 MPa at 17 °C for 10 min	12 °C, RH > 95% for 10 d, RH 80% till end of ripening.	21 d	Putrescine and cadaverine level decreased (88% and 98% reduction compared with the control)	(Latorre-Moratalla and others 2007)
Dry-cured sausage (Chorizo)	350 MPa for 15 min at 20 °C	2 °C	160 d	Decrease in tyramine (17%), putrescine (8.7%) and cadaverine (12.5%)	(Ruiz-Capillas and others 2007)
Yellowfin tuna and mahi-mahi	300 to 400 MPa for 5 min	4.4 °C	12 d	Reduced histamine producing bacteria (*Morganella morganii*) and their histidine decarboxylase activity	(Bolton and others 2009)

Treating fermented sausage with high pressure (350 MPa/15 min) reduced lactic acid bacteria (20.1%) and reduced cadaverine (12.5%), putrescine (8.7%), and tyramine (17%) levels during 160 d chilled storage compared to sausage not treated with HHP (Ruiz-Capillas and others 2007). Histamine forming bacteria and histidine decarboxylase activity in yellowfin tuna and mahi-mahi fish can be reduced by applying HHP between 300 and 400 MPa without affecting the quality of the fish (Bolton and others 2009).

HHP (300 MPa at 40 °C for 10 min) applied during Saukraut fermentation, extended the shelf life through microbial reduction (Peñas and others 2010). Although this study did not analyze biogenic amines, the product is known to contain biogenic amines (Shalaby 1996).

Overall, there is limited information on the efficacy of HHP treatment on the control of biogenic amines through the treatment of raw materials (Latorre-Moratalla and others 2007) with evidence of both increased and decreased biogenic amine formation (Novella-Rodriguez and others 2002; Latorre-Moratalla and others 2007; Ruiz-Capillas and others 2007). It is possible that HHP affects the enzymes as well as the bacteria that cause biogenic amine formation, although this aspect has not been studied.

### Irradiation

Irradiation to extend the shelf life of food was introduced in the 1950s (Mbarki and others 2009). Irradiation has been used in the food industry to prolong shelf life and ensure safety of foods, reducing the use of chemical preservatives (Loaharanu 1989; Radomyski and others 1994; Thayer 1994; Ahn and others 2002a; Ahn and others 2002b).

Irradiation may control biogenic amine formation in foods (Table 3), by radiolysis of biogenic amines (Mbarki and others 2009) and by reducing the number of bacteria responsible for biogenic amine production (Kim and others 2003). Radiolytic degradation of biogenic amines was demonstrated in a model system. Histamine, cadaverine, putrescine, spermidine, spermine, tryptamine, tyramine, and agmatine standards were irradiated at 2.5, 5, 10, 20, and 25 kGy after being dissolved in distilled water at concentrations of 100 ppm. The degradation observed was between 5 and 100%, overall showing 95% degradation of all amines at 20 kGy. Significant degradation of spermine, spermidine, and putrescine occurred above 5 kGy (Kim and others 2004). However, as the authors have noted, the study is based only on a model system, the application to a food system requires further investigation. The high dosage use may affect the sensory quality of the food. Irradiation at 10 kGy is considered safe to apply to any food product (WHO 1994), but levels higher than this require studies on the sensory characteristics and safety of treated food. Shelf life extension of food products treated with irradiation has been applied to many foods including pork and beef (Min and others 2007b), sausage (Kim and others 2005a), soybean paste (Kim and others 2003; Kim and others 2005b), chicken (Min and others 2007a), and fish (Mbarki and others 2009; Schirmer and others 2009).

**Table 3:** Biogenic amines reduction through irradiation.

**Food type**	**Irradiation condition**	**Storage condition**	**Storage time**	**Biogenic amines reduced**	**Reference**
Distilled water containing 100 ppm of biogenic amines	Applied doses: 0, 2.5, 5, 10, 15, 20, 25 kGy (best reduced at 25)	–	–	At 20 kGy putrescine, spermidine, phenylethylamine, spermine, and histamine were completely destroyed.	(Kim and others 2004)
	Source strength: 100 kCi dose rate: 5 kGy/h at 12 °C			At 25 kGy the remaining amines, cadaverine, tryptamine, tyramine and agmatine were completely destroyed.	

**Food type**	**Irradiation condition**	**Storage condition**	**Storage time**	**Reduction in the formation of biogenic amines**	**Reference**

Pepperoni sausage (fermented)	Applied doses: 0, 5, 10, 20 kGy (best reduced at 20) Source strength: 100 kCi 5 dose rate: kGy/h at 12 °C	Air packaged and stored at 4 °C	4 wk	Decreased amines at 20 kGy: putrescine (from 2.6 ppm to complete destruction), tyramine (dropped from 0.9 to 0.2 ppm), spermine (dropped from 9.6 to 4.2 ppm) and spermidine (dropped from 11.8 to 8.4 ppm)	(Kim and others 2005a)
Low-salt fermented soybean paste (with 6% and 8% salt)	Applied doses: 5, 10, 15 kGy (best reduced at 15)	25 °C	12 wk	Putrescine (dropped from 3124 to 797.3 ppm at 8% salt and 15 kGy),	(Kim and others 2005b)
	Source strength: 100 kCi				
	Dose rate: 5 kGy/h at 13 °C				
Beef and pork	Applied doses: 0, 0.5, 1, 2 kGy (best reduced at 2)Source strength: 100 kCi dose rate: 83.3 Gy/min at 12 °C	4 °C	20 h	Decreased amines at 2 Gy: putrescine (dropped from 4.7 to 2 ppm in beef, and 2.3 to 0.3 ppm in pork), tyramine, (dropped from 24.7 to 9.3 ppm in beef, and 1.3 to 0.8 ppm in pork), and spermine (dropped from 28.4 to 22.4 ppm in beef, and 31.3 to 25.9 ppm in pork)	(Min and others 2007b)
Vacuum packed Chub mackerel (*Scomber japonicus*)	Applied dose: 1.5 kGy	At 1 °C with air circulation	14 d	Significant reduction of histamine (dropped from 50.91 to 2.87 ppm)	(Mbarki and others 2009)

Ground pork and beef inoculated with *Alcaligenes faecalis, Bacillus cereus,* and *Enterobacter cloacae* were treated with gamma irradiation doses of 2 kGy. The total amount of biogenic amines (histamine, tyramine, spermidine, beta-phenylethylamine, tryptamine, cadaverine, and putrescine) formed during 24 h storage at 4 °C was reduced by the treatment (Min and others 2007b). Levels of tyramine, spermidine, spermine, and putrescine were effectively reduced in pepperoni sausage by gamma irradiation (5, 10, 20 kGy) (Kim and others 2005a). Gamma irradiation at 5, 10, or 15 kGy reduced putrescine, cadaverine, agmatine, histamine, tryptamine, spermine, and spermidine during fermentation of low-salt fermented soy paste (Kim and others 2005b). Chub mackerel (*Scomber japonicus*) in chilled storage, after irradiation followed by vacuum packing slowed the formation of biogenic amines (Mbarki and others 2009).

While irradiation delays the formation of some biogenic amines, there are reports of irradiation enhancing the formation of other biogenic amines (Kim and others 2003; Wei and others 2009). Korean fermented soybean paste treated by irradiation, did not have a significant difference in biogenic amine content compared with the control, although the concentration of histamine, tyramine, spermidine, and putrescine decreased, during fermentation. Possible explanations for the latter include a reduction of microorganisms by irradiation, or some of the preformed biogenic amines may have been utilized as substrates by microbes, during fermentation (Nout 1994; Kim and others 2003). Biogenic amines in raw chicken breast and thigh meat were reduced using irradiation at a dose of 2 kGy, even though some of the biogenic amines (histamine, spermidine, and spermine) were increased, perhaps because irradiation changes the structure and physiological properties of enzymes that form biogenic amines (Min and others 2007a). Prior to ripening, Chinese Rugoa ham was irradiated with a dose of 5 kGy, producing a degradation of spermine, putrescine, and tyramine, but formation of tyrptamine, spermidine, phenylethylamine, and cadaverine increased compared to controls after irradiation. The increase of the latter may be due to the ham being ripened after irradiation and the growth of decarboxylating microorganisms, during the ripening process (Wei and others 2009).

There is some consumer resistance to the use of irradiation, and this includes taste problems (Mbarki and others 2009). The biogenic amine reduction in foods seems to be more effective at high doses of irradiation (Mbarki and others 2008). However, high doses are most likely to result in what has been described as “irradiation taste” (Schirmer and others 2009). It may also be possible that irradiation also inhibits the decarboxylase enzyme activity; however, this requires investigation. Radiolytic products of biogenic amines in irradiated food and their biological effect need to be studied (Kim and others 2004). In summary, irradiation has potential use in delaying biogenic amine accumulation, but the method requires further study.

### Packaging

Preservation through packaging usually involves changing the gaseous mixture of the environment surrounding the product. This may delay the production of biogenic amines, due to inhibition of the microorganisms or the enzymes producing biogenic amines. The histidine decarboxylase enzyme was reported to be more effective in the absence of oxygen (O_2_), while histaminases (such as DAO), the enzyme that oxidizes histamine, were found effective, only in the presence of O_2_ (Kapeller-Adler 1941). However, both anaerobic and aerobic bacteria are capable of producing biogenic amines, and as well as degrading biogenic amines so finding a balance that will control microbial growth and enzyme activity may be difficult.

There are reports on the successful control of biogenic amines through packaging (Table 4). These include vacuum packaging of salmon (Mbarki and others 2009), MAP of fish (Emborg and others 2005; Dalgaard and others 2006), chicken (Balamatsia and others 2006; Patsias and others 2006), sausage (Kim and others 2005a), and active packaging of seer fish (Mohan and others 2009).

**Table 4:** Biogenic amines reduction through packaging.

**Food type**	**Packaging condition**	**Storage condition**	**Storage time**	**Reduction in the formation of biogenic amines**	**Reference**
Yellowfin tuna (*Thunnus albacares*)	MAP (40% CO_2_/60% O_2_)	1 °C	28 d	No histamine formed/strong inhibitory effect to histamine production and growth of *Morganella morganii* and *Photobacterium phosphorium*	(Emborg and others 2005)
Garfish	MAP (40% CO_2_ and 60% N_2_)	0 and 5 °C	38 d	Reduced histamine formation in thawed MAP garfish	(Dalgaard and others 2006)
Breast chicken meat	MAP (30% CO_2_, 70% N_2_)	4 °C	17 d	Slight decrease in cadaverine (223.7 ppm in MAP and 252.7 ppm in air packaging) and putrescine (354 ppm in MAP and 409.6 ppm in air packaging) compared to air packaging	(Balamatsia and others 2006)
Precooked chicken meat	MAP (30% CO_2_, 70% N_2_)	4 °C	23 d	Reduced putrescine (90.4 ppm under MAP at 23rd d, 202.6 ppm at 23rd d under air) and tyramine (8.8 ppm under MAP at 23rd d, 18.8 ppm at 23rd d under air)	(Patsias and others 2006)
Chub mackerel (*Scomber japonicus*)	Vacuum packaged	1 °C	7 d	Slight reduction (on 7th d of storage), of biogenic amines; histamine (dropped from 57.22 to 47.66 ppm), cadaverine (dropped from 18.93 to 10.07 ppm), spermidine (dropped from 10.29 to 6.94 ppm), putrescine (dropped from 21.13 to 13.52 ppm)	(Mbarki and others 2009)
Seer fish (*Scomberomorus commerson*)	Packed in pouches (a multilayer film of ethylene-vinyl alcohol) with O_2_ scavenger sachets	0 to 2 °C	30 d	Delayed formation of putrescine (on 15th d in air pack contained 14.62 ppm and on 30th d fish held in O_2_ scavenger pack contained 11.1 ppm); cadaverine (on 15th d in air pack contained 14.77 ppm and 2.16 ppm in O_2_ scavenger pack); histamine (air pack reached 6.9 ppm on the 15th d storage while the same level reached on the 30th d in O_2_ scavenger pack); tyramine (1.7 ppm on 15th day in air pack and 0.07 ppm on 30th d in O_2_ pack)	(Mohan and others 2009)

In active packaging, different gas scavengers are used (O_2_, carbon dioxide [CO_2_]) to control the environment within the pack. O_2_ scavengers eliminate O_2_ in the headspace and product < 0.01% (Mohan and others 2009). Mohan and others (2009) found that the presence of O_2_/air increased biogenic amine production in Seer fish (*Scomberomorus commerson*) steaks and by removing O_2_ (99%) with O_2_ scavengers (active packaging), biogenic amines in the fish were lower and shelf life was extended from 12 d (air) to 20 d. Biogenic amine producers were apparently from aerobic bacteria that possess the decarboxylase activity, thus removal of O_2_ inhibited aerobic bacteria and delayed biogenic amine accumulation.

Vacuum packaging extends the shelf life of food compared to air packaging (González-Montalvo and others 2007). Recently, a novel packaging method was developed (Schirmer and others 2009) that involves combining organic acids with CO_2_ from the headspace dissolving into the product until a vacuum is formed (“CO_2_-vacuum packed” products). This was used on salmon as an effective method to inhibit microbial growth and extend shelf life. Microbes that were reduced included *Photobacterium phosphorium* that has been reported as an active histamine former able to grow under normal MAP conditions producing more than 1000 ppm histamine (Kanki and others 2004; Emborg and others 2005; Dalgaard and others 2006; Tao and others 2009).

MAP extends the shelf life of food longer than vacuum packing (Özogul and others 2004). Histamine content in vacuum packed tuna was >7000 ppm, and the bacteria responsible for were suspected to be either *P. phosphoreum* or *M. morganii—M. psychrotolerans.* Histamine production was controlled when MAP with a gas mix of 40% CO_2_/60% O_2_ was applied to tuna stored for 28 d at 1.0 °C (Emborg and others 2005). This method may have controlled histamine formation by the inhibition of the growth of the psychrotrophic histamine producing bacteria *P. phosphoreum* and *M. morganii*–*M. psychrotolerans*. Therefore, it was suggested to use MAP with the above gas mixture for lean fish, such as tuna loins, to avoid possible scombroid poisoning.

Dalgaard and others (2006) demonstrated the synergistic effect of MAP with a gas mixture of 40% CO_2_/60% N_2_ and freezing and thawing to control histamine production in chilled garfish by *P. phosphoreum* that had produced histamine >1000 ppm at chilled storage under air and MAP. When the garfish was frozen, thawed and stored at 5 °C, the shelf life was 70% longer under the MAP gas mix and histamine production was reduced compared with storage in air. The authors considered that this was because the *P. phosphoreum* responsible for histamine production was inactivated by freezing and thawing (Dalgaard and others 2006). MAP cod fillets with 500 ppm Na_2_CaEDTA (antimicrobial) reduced *P. phosphoreum* by 40% and extended shelf life by 40% at 0 °C (Dalgaard and others 1998). Since *P. phosphoreum* is able to form >1000 ppm histamine below 5 °C (Dalgaard and others 2006), this method may reduce histamine content in cod through the inhibition of the bacteria.

Chicken breast meat stored under MAP (30% CO_2_/70% N_2_) was evaluated for shelf life up to 17 d at 4 °C (Balamatsia and others 2006). On the 17th d of storage, the histamine level only reached 26.8 ppm, the delay may be due to specific types of histamine producing bacteria, such as *Enterobacteriaceae* having difficulty growing under MAP and when the total bacterial level reached 10^7^ cfu/g on the 11th d of storage, the histamine was detected (5.4 ppm). However, cadaverine and putrescine in chicken under MAP was only slightly less (223.7 ± 12.0 ppm and 354.0 ± 17.2 ppm, respectively) than chicken breast meat in aerobic packaging (252.7 ± 12.8 ppm and 409.6 ± 18.4 ppm, respectively). Cadaverine and putrescine may be reduced under MAP, due to lactic acid bacteria, that may either utilize these amines as substrates or may have oxidizing enzymes that degraded these amines or delayed the accumulation. Some lactic acid bacteria have been shown to degrade biogenic amines (Dapkevicius and others 2000).

Patsias and others (2006) studied precooked chicken meat under air and MAP (30% CO_2_/70% N_2_) at 4 °C for up to 23 d. When the biogenic amines levels were compared after 23 d of storage under MAP, putrescine and tyramine were reduced compared with packaging under air.

Kim and others (2005a) found the use of MAP with a gas mixture of 25% CO_2_/75% N_2_ gas did not reduce the production of biogenic amines in pepperoni. Other packaging types, air and vacuum were found more effective than MAP, although in general, each packaging type had a different affect on individual amines. The effect of MAP on the suppression of biogenic amine formation in pepporini requires more studies using different gas mixtures such as those used by Dalgaard and others (2006) on fish and Patsias and others (2006) on chicken.

In summary, compared to air packaging, active packaging, vacuum packaging, and MAP inhibit or delay formation of biogenic amines more effectively, through inhibition of biogenic amines forming bacteria or enzyme activity, but the success of inhibition largely depends on the type of microflora, and it is environmental conditions such as temperature, and also the gas mix used in case of MAP. It may also be product specific.

### Microbial modeling

Microbial modeling can be used to study the growth and inactivation of microorganisms (Zwietering and others 1990; Xiong and others 1999; Van Boekel 2002) with the aim of controlling growth and predicting risk factors (Ross 1996; Neumeyer and others 1997; Seo and others 2007). Modeling microorganisms responsible for biogenic amine formation (Emborg and Dalgaard 2008a, 2008b; Gardini and others 2008) has been used to explore options for biogenic amine control.

Temperature, time, and pH affect biogenic amine production, and these could be modeled for particular microbial species in specific foods. Such models may help design conditions to limit amine production. However, the draw back of this method is that there are many known bacterial species capable of producing biogenic amines already known and probably others yet to be found therefore generic modeling to account for all these species would be complex, time consuming, and tedious. Currently available models for biogenic amine producing bacteria include those of Emborg and Dalgaard (2008a) on *M. psychrotolerans* and *M. morganii* in fish, and Gardini and others (2008) for *Enterococcus faecalis* EF37 in sausage.

Emborg and Dalgaard (2008a) developed a mathematical model for the histamine forming bacteria, *M. psychrotolerans* and *M. morganii* and identified the conditions to inhibit the growth of these bacteria through heat in canned tuna meat, thawed garfish meat, tuna juice and broth. The mathematical equations of the model (equations 1 to 3) have subsequently been incorporated into freely available software (Dalgaard 2009). The main parameter was the effect of temperature on the growth and inactivation of *M. psychrotolerans* and *M. morganii*.
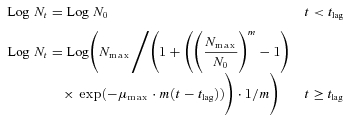
(1)
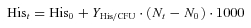
(2)
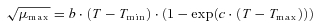
(3)where μ_max_= maximum specific growth rate, *N*_max_= the maximum cell density, *Y*_His/CFU_= yield factor for histamine formation, *N*_0_= actual initial concentration, *t*_lag_= 2.55 ln(2)/μ_max_= lag time, *N_t_*= cell concentration at time *t*, *m*= parameter to characterize growth dampening when the cell concentration *N_t_* approaches the maximum cell concentration (*N*_max_), His*_t_* and His_0_= concentration of histamine (ppm) at time t and 0 and *N_t_* and *N*_0_(cfu/g or cfu/mL) = corresponding cell concentration, *b* and *c*= constants, *T*= temperature, and *T*_min_ and *T*_max_= the theoretical minimum and maximum temperatures, respectively (Emborg and Dalgaard 2008a p. 236).

The model predicted the time for histamine to reach 100 ppm was longer than the observed value and to reach 500 and 2000 ppm was shorter than the observed value. Therefore, the model could be used to help control the formation of histamine in food to <500 ppm, but not down to 100 ppm (Emborg and Dalgaard 2008a). Emborg and Dalgaard (2008b) also modeled the growth of the histamine forming bacteria, *M. psychrotolerans* examining the effects of pH, water activity, temperature, and CO_2_. The model detailed histamine formation and the growth of bacteria under different storage conditions and different product characteristics (NaCl, water activity, pH) of tuna meat. The parameters included in the model were temperature (0 to 20 °C), atmosphere (0 to 100% CO_2_), pH (5.4 to 6.5), and NaCl (0.0 to 6.0%). This model provided a slightly conservative (fail-safe) prediction for the time when the histamine concentration is toxic, between 500 and 2000 ppm, even though the predictions were not very accurate. The kinetic approach used in this model maybe used to develop models for other histamine forming bacteria in different storage conditions and with different fish (Emborg and Dalgaard 2008b).

Gardini and others (2008) modeled *E. faecalis EF37* for biogenic amine formation in dry fermented sausages. Fermentation was carried out for 30 d, and samples were analyzed on days 3, 5, 19, and 30. The parameters included were glucose (0, 700, 1400 ppm), temperature (15, 20, 25 °C), and NaCl (0, 2.5, 5%). The *Enterococci* count reached 10^5^ cfu/g after 30 d fermentation in all the conditions. NaCl at 5% reduced tyramine to a negligible amounts (<1 ppm), while the tyramine level with 0% NaCl was >200 ppm. The authors found that the most important variable in preventing tyramine formation was the salt content. Temperature and glucose had negligible effects on tyramine accumulation. The level of tyrosine decarboxylase (tdc) decreased, when NaCl levels were >2%. The tdc activity increased with an increase in temperature (20 to 25 °C) (Gardini and others 2008).

In summary, mathematical modeling has been used on biogenic amine forming bacteria: *M. morgani*, *M. psychotolerans,* and *E. faecalis*. Parameters studied included water activity, temperature, salt content, pH, glucose, and CO_2_. For *E. faecalis* growth and activity in sausage, >2% salt content decreased biogenic amines. A correlation was found between the tdc level and tyramine formation: that is as tdc increased the tyramine level also increased during the fermentation of sausage. Since limited modeling on biogenic amine forming bacteria has been reported, there is an opportunity to develop new models or improve current models through further studies.

## Starter Cultures

Starter cultures used in fermentation can also delay the formation of biogenic amines (Bover-Cid and others 2001b, 2001c; Spicka and others 2002; Latorre-Moratalla and others 2007; Mah and Hwang 2009b). Starters used for fermented foods are either amine-negative (not able to decarboxylate amino acid into biogenic amines) or amine oxidizing (oxidize biogenic amines into aldehyde, hydroden peroxide, and ammonia) bacteria (Bover-Cid and others 2000a; Suzzi and Gardini 2003). These bacteria require optimal growth conditions to dominate over biogenic amine producing (Xu and others 2010) and other contaminant bacteria (Maijala and others 1995a; Maijala and others 1995b; Hu and others 2008). Typical fermented foods where the effect of starters on biogenic amines have been studied include sausages (Bover-Cid and others 2000a; Bover-Cid and others 2000b; Latorre-Moratalla and others 2007), cabbage (Spicka and others 2002), cheese (Fernandez-García and others 2000; Nieto-Arribas and others 2009), wine (Hernández-Orte and others 2008), and vegetables (Tamang and others 2009).

A number of bacteria have been found to have negative decarboxylase activity or possess enzymes that oxidize biogenic amines in food (amine-negative bacteria) Artisanal Manchego cheese isolates, of *Lactobacillus plantarum* and *Lactobacillus paracasei subsp. paracasei* were found to be amine-negative bacteria except for one isolate from the latter, found producing tyramine. These amine-negative organisms were suggested as potential starters for cheese production (Nieto-Arribas and others 2009). Amine-negative starters, *Staphylococcus xylosus* and *Lactobacillus carvatus* delay putrescine and cadaverine formation during the ripening and storage of dry fermented sausages (Bover-Cid and others 2001b). The inoculation of amine-negative mixed starters, *Pediococcus acidilactici*, *Staphylococcus carnosus*, *Lactobacillus sake*, *S. xylosus* into cold smoked fish, can help control biogenic amines (Petäjä and others 2000). Amine-negative mixed starters of *S. carnosus*, *Lactobacillus sakei*, and *S. xylosus* have also been used during the fermentation of dry sausage and were found to suppress biogenic amine accumulation (Bover-Cid and others 2001b). Mixed starters of *L. plantarum*, *Pediococcus pentosaceus*, *S. xylosus*, *Lactobacillus casei* inhibited formation of biogenic amines and suppressed the contaminant microorganisms in silver carp sausages (Hu and others 2007).

Mixed starters produce a synergistic effect in the control of biogenic amines (Hu and others 2007). The use of mixed starters results in a large pH decrease (Hu and others 2007) that may be an additional factor contributing to reducing biogenic amine accumulation.

Effective control of biogenic amines may require a combination of several factors (Latorre-Moratalla and others 2010). For example, the control of biogenic amines with starters is likely to be most effective with good quality raw material (Bover-Cid and others 2000b; Petäjä and others 2000; Hu and others 2007).

### Methods for oxidizing/degrading formed biogenic amines

Even though many methods are available, as described above, for delaying biogenic amine accumulation, few methods are available for degrading biogenic amines. Such methods include the use of oxidizing microorganisms, such as biogenic amine oxidizing bacteria, and enzymes such as DAO. Biogenic amine degrading bacteria could be introduced into a food processing step to degrade the biogenic amines in the food, or the bacteria could be used as a starter for fermented foods. Bacteria described as biogenic amine oxidizers include *Micrococcus varians* (Leuschner and Hammes 1998b), *Natrinema gari* (Tapingkae and others 2010) *Brevibacterium linen* (Leuschner and Hammes 1998a), *Vergibacillus sp* SK33 (Yongsawatdigul and others 2007), *L. sakei*, *Lactobacillus curvatus* (Dapkevicius and others 2000), and *S. xylosus* (Mah and Hwang 2009b). *Arthrobacter crystallopoietes* KAIT-B-007 contains the amine oxidizing enzyme (DAO) that is specific to histamine oxidation. Although this enzyme was isolated and the activity studied (Sekiguchi and others 2004), the source bacteria have not been studied for biogenic amine degradation in food. *Micrococcus Varians,* having tyramine oxidase, degraded tyramine during sausage fermentation (Leuschner and Hammes 1998b). *Natrinema gari*, an extremely halophilic archaea isolated from anchovy fish sauce, was reported to degrade histamine in high-salt media. The optimum temperature and pH for the degradation was between 6.5 and 8.3 and 40 and 55 °C, respectively, and the NaCl concentration was 3.5 to 5 M (Tapingkae and others 2010). However, no studies of this bacterium have been done in food. *Brevibacterium linen* reduced histamine by 70% and tyramine by 55% in Munster cheese over 4 wk of ripening (Leuschner and Hammes 1998a). Mah and Hwang (2009b) studied biogenic amine reduction in Myeolchi-jeot, a salted and fermented anchovy (*Engraulis japonicas*) by applying starter cultures during ripening. *Staphylococcus xylosus* No. 0538 degraded histamine and tyramine by 38% and 4%, respectively, and the total biogenic amine level was decreased by 16%.

The histamine level in fish sauce has been regulated in Canada and USA, with the maximum allowable limit set at 200 ppm and 500 ppm, respectively (Brillantes and others 2002). Per meal, an intake of 40 mg of biogenic amines is considered toxic (Nout 1994). Histamine poisoning incidents due to fish sauce may have occurred but may not be reported as the symptoms are similar to those of food allergies (Tsai and others 2006). Histamine content in Thai fish sauce ranges between 200 and 600 ppm (Brillantes and Samosorn 2001). Inoculation of 10% (w/w) *Virgibacillus sp.* SK33 in Thai fish sauce fermentation, reduced histamine production by 50% (117.6 ± 0.07 ppm) from an initial level of 215.3 ± 4.41 ppm histamine (Yongsawatdigul and others 2007). However, the tyramine level increased from an initial level of 49.6 ± 0.93 ppm to 90.6 ± 1.45 ppm in both the control and in the fish sauce containing the starter culture, respectively. The reasons for the increase in tyramine need to be determined as do the effects of other factors such as NaCl, pH, and temperature on the biogenic amine content of fermented fish sauce (Yongsawatdigul and others 2007). It is possible that *Virgibacillus sp.* SK33 may also be used successfully to control biogenic amines in other foods.

*Lactobacillus spp.* are also able to reduce biogenic amines. Dapkevicius and others (2000) studied the ability of lactic acid bacteria isolated from mackerel fish paste to degrade biogenic amines. Five cultures (*L. sakei* 15.05, *L. sakei* 15.18, *L. sakei* 15.36, *L. sakei* 15.39, and *L. curvatus* 15.35) were found to degrade histamine (20 to 54%) in deMan, Rogosa and Sharpe (MRS) broth containing 50 ppm histamine, and 2 cultures (*L. sakei* 15.18, and *L. sakei* 15.36) degraded histamine (50 to 54%) in the fish slurry (containing 10 ppm histamine) (Dapkevicius and others 2000). *Lactobacillus sakei* 15.18 and *L. sakei* 15.36 are potential starters to degrade histamine during food fermentation. DAO was also studied by the same authors for the potential for histamine degradation in broth and fish slurry.

DAO is another option for biogenic amine degradation. The ability of DAO to degrade histamine in both phosphate buffer (pH 7.0), and ensiled fish slurry (pH 4.5) was studied (Dapkevicius and others 2000). DAO was investigated by applying the similar conditions found in fish silage to fish slurry; 2% NaCl, 12% sucrose, 0.05% cysteine. DAO degraded histamine (approximately 40% compared with a control) in fish slurry incubated at 30 °C with starting pH of 6.4. There was no effect on histamine degradation by DAO with 12% sucrose and 2% NaCl. The addition of 0.05% cysteine decreased histamine degradation and degradation did not occur at pH 4.5. The optimum temperature for DAO activity is 37 °C. DAO activity needs to be investigated in a variety of foods to determine the effectiveness of the enzyme in degrading biogenic amines in different food matrices. A factorial designed experiment combining key factors such as temperature, pH, and DAO concentration on the degradation of biogenic amines in food will be useful in recommending DAO for use in specific foods.

The use of bacteria with amine oxidizing activity or oxidizing enzymes to reduce biogenic amine levels in foods is a potential control measure where it is difficult to control biogenic amine levels through the traditional means of refrigeration, and to eliminate already formed biogenic amines in food.

## Conclusion

The existing method for controlling biogenic amines in food is refrigeration. However, since some bacteria that form biogenic amines can grow below 5 °C, refrigeration alone is not always controlling biogenic amines and thus emerging control measures need to be considered. Emerging control measures for delaying biogenic amine formation include HHP, irradiation, packaging, microbial modeling, and the use of food additives or preservatives. These methods only delay biogenic amines formation in food primarily through the inhibition of bacteria or the decarboxylase enzyme activity responsible for amine production. Application of sufficient heat or freezing storage can prevent further development of biogenic amines, although product needs to be protected from recontamination in the case of heat and from thawing in the case of freezing.

Refrigeration is not always a feasible option for artesian fishers, thus the microbiologically stable product having high biogenic amines need to be controlled by other means. The use of amine oxidizing bacteria and enzymes are the best options.

There are some practical limitations on the use of some of these methods depending on the resources available. The use of the more novel emerging methods and combinations of control measures, often described as hurdle technology, for the control of biogenic amines needs to be further investigated.

## Nomenclature

DAO: diamine oxidase
MAP: modified atmosphere packaging
NOAEL: no observed adverse effect level
MAOI: monoamine oxidase inhibitors
HHP: high hydrostatic pressure
NaCl: sodium chloride
GDL: glucono-delta-lactone
